# Structural Investigation of the Oligosaccharide Portion Isolated from the Lipooligosaccharide of the Permafrost Psychrophile *Psychrobacter arcticus* 273-4

**DOI:** 10.3390/md13074539

**Published:** 2015-07-22

**Authors:** Angela Casillo, Ermenegilda Parrilli, Sannino Filomena, Buko Lindner, Rosa Lanzetta, Michelangelo Parrilli, Maria Luisa Tutino, Maria Michela Corsaro

**Affiliations:** 1 Dipartimento di Scienze Chimiche, Università degli Studi di Napoli Federico II, Complesso Universitario Monte S. Angelo, Via Cintia 4, Napoli 80126, Italy; E-Mails: angela.casillo@unina.it (A.C.); erparril@unina.it (E.P.); filomena.sannino2@unina.it (S.F.); lanzetta@unina.it (R.L.); tutino@unina.it (M.L.T.); 2 Institute of Protein Biochemistry, CNR, Via Pietro Castellino 111, Napoli 80131, Italy; 3 Division of Bioanalytical Chemistry, Research Center Borstel, Leibniz-Center for Medicine and Biosciences, Parkallee 10, BorstelD-23845, Germany; E-Mail: blindner@fz-borstel.de; 4 Dipartimento di Biologia, Università degli Studi di Napoli Federico II, Complesso Universitario Monte S. Angelo, Via Cintia 4, Napoli 80126, Italy; E-Mail: parrilli@unina.it

**Keywords:** *Psychrobacter arcticus* strain 273-4, glycoconjugates, lipopolysaccharide, *N*-acetylmuramic acid, structural determination, NMR spectroscopy

## Abstract

Psychrophilic microorganisms have successfully colonized all permanently cold environments from the deep sea to mountain and polar regions. The ability of an organism to survive and grow in cryoenviroments depends on a number of adaptive strategies aimed at maintaining vital cellular functions at subzero temperatures, which include the structural modifications of the membrane. To understand the role of the membrane in the adaptation, it is necessary to characterize the cell-wall components, such as the lipopolysaccharides, that represent the major constituent of the outer membrane. The aim of this study was to investigate the structure of the carbohydrate backbone of the lipooligosaccharide (LOS) isolated from the cold-adapted *Psychrobacter arcticus* 273-4. The strain, isolated from a 20,000-to-30,000-year-old continuously frozen permafrost in Siberia, was cultivated at 4 °C. The LOS was isolated from dry cells and analyzed by means of chemical methods. In particular, it was degraded either by mild acid hydrolysis or by hydrazinolysis and investigated in detail by ^1^H and ^13^C NMR spectroscopy and by ESI FT-ICR mass spectrometry. The oligosaccharide was characterized by the substitution of the heptose residue, usually linked to Kdo in the inner core, with a glucose, and for the unusual presence of *N*-acetylmuramic acid.

## 1. Introduction

Cold environments are arguably the most widespread on our planet and in our solar system [1]. At least 80% of terrestrial habitats and oceans are permanently cold, together with six of the other eight planets of our solar system. Hence, understanding life’s adaptation to cold environments on our planet could be useful in the search for and understanding of life on other planets [2].

Many microorganisms populate Arctic and Antarctic regions [3], and those inhabiting permafrost in particular are good candidates to study cold-adaptation, due to the mean annual temperature between −10 and −12 °C in the Arctic and between −18 and −27 °C in the Antarctic [4]. Although living microorganisms can be successfully recovered either from ice or permafrost, the latter is a more proficient environment to sustain longer growth time due to its heterogeneous soil particles and larger reservoirs of nutrients [5,6,7].

One physiological response to the cold environment is the alteration of membrane components, such as the presence of unsaturated and branched fatty acids in phospholipids that maintain membrane fluidity [8], and the different phosphorylation of membrane proteins and lipopolysaccharides [9,10,11,12,13,14].

The lipopolysaccharides (LPSs) are the major component of the outer membrane (OM) of almost all Gram-negative bacteria and of some cyanobacteria [15,16,17,18], constituting approximately 75% of the outer surface. The LPSs are heat-stable amphiphilic molecules indispensable for the viability and survival of Gram-negative bacteria, as they heavily contribute to the structural integrity of the OM and to the protection of the bacterial cell envelope [19].

The structure of an intact smooth (*S*)-type bacterial LPS molecule can be divided into three covalently linked domains: the glycolipid anchor, called lipid A, the intermediate core oligosaccharide (core), and the *O*-specific polysaccharide (*O*-chain) [20]. However, the rough (*R*)-type LPSs (also called lipooligosaccharides, LOSs) are completely devoid of the *O*-specific polysaccharide chain either due to genetic mutation or the inherent nature of bacteria [21].

Extreme habitats drive microbial components to fulfill cell homeostasis through the maintenance of membrane integrity. Thus, the structural characterization of LPSs of cold-adapted Gram-negative bacteria grown at low temperatures could give insight into the cryo-adaptation phenomena understanding.

Until now, only LPSs from marine Arctic [11,22] and Antarctic [12,23] Gram-negative microorganisms have been characterized, but very little is known about isolates from permafrost. It has been shown that viable bacteria are abundant in Siberian permafrost [6,24], and the most frequently isolated from the Kolyma permafrost of northeast Siberia include *Arthrobacter*, *Exiguobacterium*, *Flavobacterium*, *Sphingomonas*, and *Psychrobacter* [4,5,6]. *Psychrobacter* is considered an indicator genus for permafrost and other polar environments [25], suggesting that many of its members are adapted to low temperatures and have evolved molecular-level changes that aid survival at low temperatures.

*Psychrobacter arcticus* 273-4 is a Gram-negative bacterium isolated from a 20,000-to-30,000-year-old continuously frozen permafrost horizon in the Kolyma region in Siberia that was not exposed to temperatures higher than 4 °C during isolation [5].

In this paper, we report the structural characterization of the carbohydrate backbone of the LOS of *Psychrobacter arcticus* 273-4 grown at 4 °C.

The lipooligosaccharide was degraded both by mild hydrazinolysis (*O*-deacylation) and by acetic acid hydrolysis. The products were investigated by means of chemical analysis, by ^1^H and ^13^C NMR spectroscopy and by electrospray ionization Fourier transform ion cyclotron resonance mass spectrometry (ESI FT-ICR MS).

## 2. Results and Discussion

### 2.1. LPS Extraction and Purification

**Figure 1:**
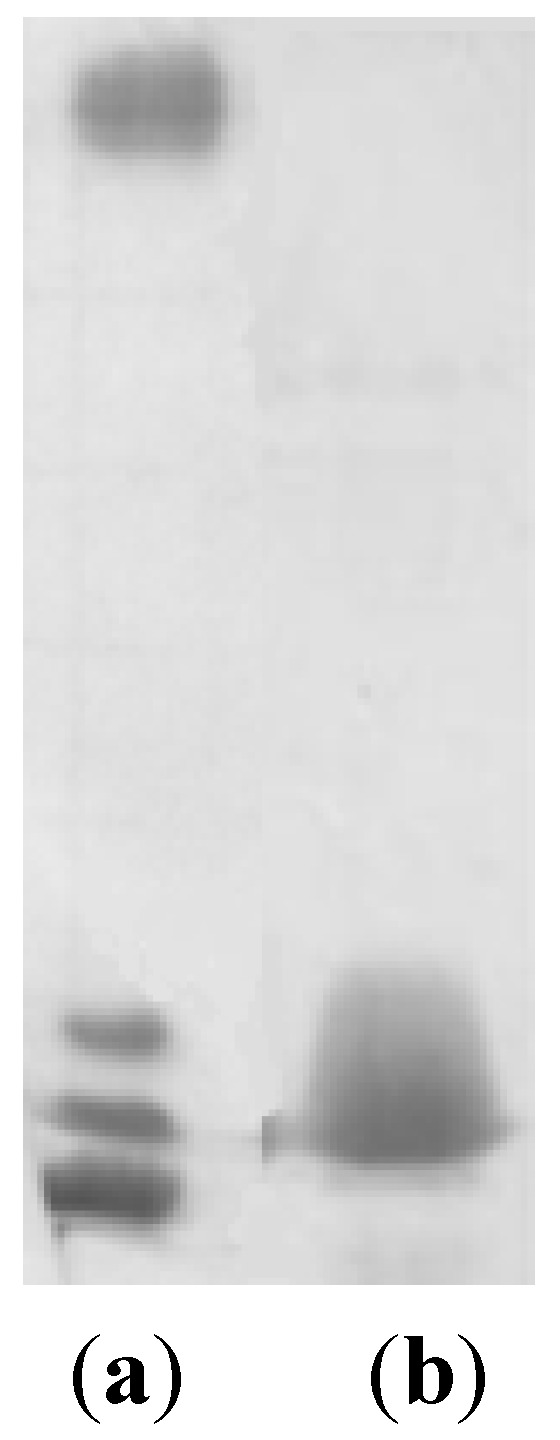
Analysis of the LPS_W_ (Lane **b**) fraction from *P. arcticus* strain 273-4 by14% DOC-PAGE. The gel was stained with silver nitrate and was compared with LPS from *E. coli* O127: B8 (Lane **a**).

*Psychrobacter arcticus* strain 273-4 cells were grown at 4 °C and removed from the medium by centrifugation. Dried bacteria cells were extracted using a phenol/chloroform/light petroleum (PCP) mixture to obtain the crude LPS. Due to the very low amount of LPS_PCP_ (0.03%), cells were extracted by phenol/water method, and the aqueous phase was dialyzed and freeze-dried. In order to purify LPS_w_ from other cell contaminants, the sample was treated with DNase, RNase, and protease followed by dialysis (LPS_W_, 3.1%). The purified sample (LPS_W_) was analyzed by DOC-PAGE electrophoresis, and the silver nitrate staining showed bands at low molecular masses, thus revealing a rough LPS (LOS, Figure 1).

The sugar composition of the intact LOS was obtained by GC-MS analysis of the acetylated methyl glycosides and revealed the occurrence of rhamnose (rha), galactose (gal), glucose (glc), *N*-acetylmuramic acid (NAM), and 3-deoxy-d-*manno*-oct-2-ulosonic acid (Kdo). Methylation analysis indicated the presence of 3-substituted Rha, terminal Glc, 4-substituted Glc, 3-substituted Gal, terminal NAM, 3,4,6-trisubstituted Glc, 3,4-disubstituted Glc, terminal Kdo, and 4,5-disubstituted Kdo. The methylation data also revealed a pyranose ring for all the residues. The absolute configurations of the sugar residues were determined by GC-MS analysis of the corresponding acetylated 2-octyl glycosides; all the hexoses were founded to be in the d-configuration, while rhamnose residue in the l-configuration. The absolute configuration of *N*-acetylmuramic acid was supported by the NMR data (see below).

Fatty acids analysis revealed the presence of the following main components: 3-hydroxy dodecanoic 12:0(3OH), 3-hydroxy tetradecanoic 14:0(3OH), tetradecanoic 14:0, tetradecenoic 14:1, pentadecanoic 15:0, and pentadecenoic 15:1 acids.

### 2.2. Deacylation of the LPS

**Figure 2:**
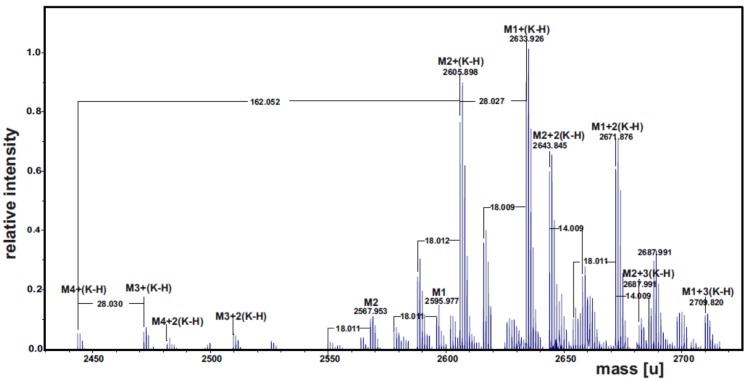
Charge deconvoluted ESI FT-ICR mass spectrum of the LOS-OH fraction isolated from *P. arcticus* 273-4. The spectrum was acquired in the negative ion mode.

The LOS_W_ was *O*-deacylated with anhydrous hydrazine and the product obtained (LOS-OH) was analyzed by ESI FT-ICR mass spectrometry. The charge deconvoluted mass spectrum showed various K-adducts [M + *n*(K − H)] of four main ion populations M1–M4 (Figure 2), the composition of which is reported in Table 1. The most abundant ion population with a mass of 2633.927 u was attributed to the following composition: DeoxyHexHex_5_Kdo_2_NAMHexN_2_P_2_ [14:0(3OH)] [12:0(3OH)] ([M1 + (K − H)], calculated monoisotopic mass: 2633. 934 u). The signal of M3, occurring at 162.052 u lower than M1, suggested the presence of ion populations containing one hexose less. In addition, the intensity of the signal of M3 suggests very low abundance of this glycoform. The ion populations M2 and M4 were attributed the same sugar composition as M1 and M3, respectively, whereas the mass difference of 28.03 u is due to a 3-hydroxy dodecanoic in place of the 3-hydroxy tetradecanoic acid.

In addition, the methylation data revealed that the lack of the hexose residue for the ion populations M3 and M4 was from the position *O*-6 of the 3,4,6-trisubstituted glucose.

**Table 1:** Composition of the main species observed in the charge deconvoluted ESI FT-ICR mass spectrum of the *O*-deacylated LOS from *P. arcticus* 273-4. Mass numbers given refer to the monoisotopic masses.

Species	Observed Mass [u]	Calculated Mass [u]	Composition ^a^
M1-H + K	2633.926	2633.934	NAMDeoxyHexHex_5_Kdo_2_HexN_2_P_2_ [14:0(3OH)] [12:0(3OH)]
M2-H + K	2605.898	2605.903	NAMDeoxyHexHex_5_Kdo_2_HexN_2_P_2_ [12:0(3OH)] [12:0(3OH)]
M3-H + K	2471.875	2471.882	NAMDeoxyHexHex_4_Kdo_2_HexN_2_P_2_ [14:0(3OH)] [12:0(3OH)]
M4-H + K	2443.845	2443.851	NAMDeoxyHexHex_4_Kdo_2_HexN_2_P_2_ [12:0(3OH)] [12:0(3OH)]

^a^ All molecular species were revealed as K salts.

### 2.3. Mild Acid Hydrolysis of the LPS

**Figure 3:**
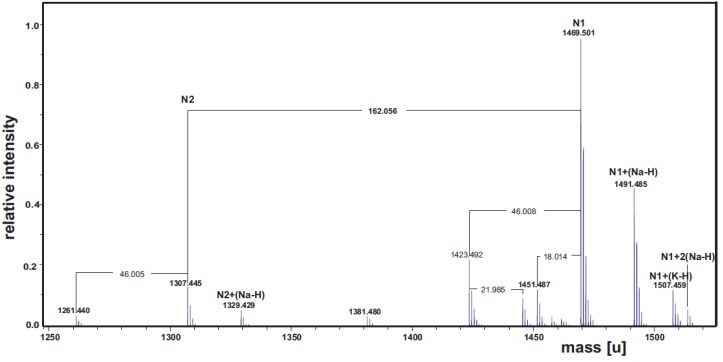
Charge deconvoluted ESI FT-ICR mass spectrum of the supernatant of acetic acid hydrolysis of *P. arcticus* 273-4 LOS. The spectrum was acquired in the negative ion mode.

The well-known ability of the LOS to form micellar aggregates in aqueous solution did not allow the direct structural NMR analysis. Thus, the LOS was hydrolyzed under mild acidic conditions to cleave the unstable Kdo glycosidic linkage between the lipid A and the saccharidic region. After centrifugation, the supernatant containing the core oligosaccharidic portion of the LOS was separated from a precipitate constituted by the lipid A. The supernatant was analyzed by ESI FT-ICR MS. The charge deconvoluted mass spectrum displayed the presence of two main ion populations (N1 and N2, Figure 3). As expected, for the most abundant N1, occurring at 1469.501 u (calculated monoisotopic mass: 1469.48 u), it was found the following composition: NAMDeoxyHexHex_5_Kdo_1_. Again, the difference of 162.056 u with N2 confirmed the presence of an ion population lacking one hexose residue. No peaks with two Kdo residues were found, since the ketosidic bond is much more acid-labile than the common aldosidic bonds. Signals at 46.00 and 18.01 u lower mass values with respect to N1 were both assignable to Kdo artifacts [26].

The supernatant mixture was further purified on a Bio-Gel P-10 chromatography column (Bio-Rad Laboratories S.r.l, Milano, Italy ), using pyridinium acetate buffer as eluent. The main obtained fraction, named OS, was studied by two-dimensional NMR spectroscopy.

### 2.4. NMR Spectroscopic Analysis of OS

To characterize the core oligosaccharide OS fraction, it was analyzed by one- and two-dimensional NMR spectroscopy. In particular, ^1^H-^1^H DQF-COSY (double quantum-filtered correlation spectroscopy), ^1^H-^1^H TOCSY (total correlation spectroscopy), ^1^H-^1^H ROESY (rotating-frame nuclear Overhauser enhancement spectroscopy), ^1^H-^13^C DEPT-HSQC (distortionless enhancement by polarization transfer-heteronuclear single quantum coherence), and ^1^H-^13^C HMBC (heteronuclear multiple bond correlation) experiments were performed.

**Figure 4:**
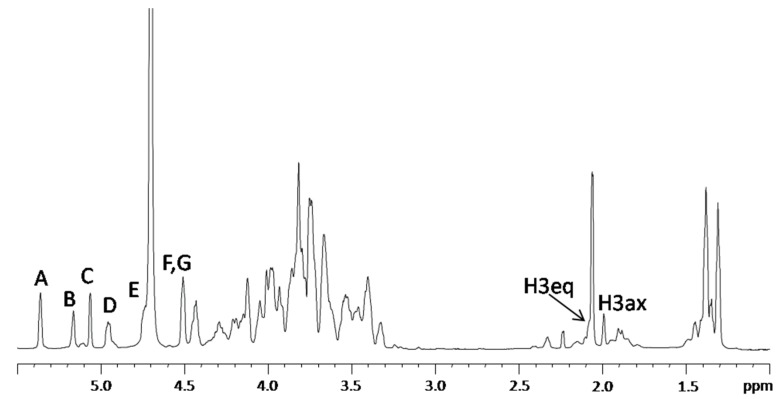
^1^H NMR spectrum of the core oligosaccharide (OS) obtained by mild hydrolysis of LOS. The spectrum was recorded in D_2_O at 310 K at 600 MHz. The letters refer to the residues as described in Table 2 and Scheme 1.

The ^1^H-NMR spectrum of the OS fraction, recorded at 310 K, is shown in Figure 4. Seven anomeric proton signals (A–G), attributable to core monosaccharide residues, were present in the region between δ 4.5 and δ 5.4 ppm (Table 2).

The ^1^H-NMR spectrum of OS was also recorded at 318 K (data not shown) in order to reduce the anomeric signals overlapping. In this experiment, the anomeric proton signal of E was clearly visible. Moreover, the integration of all anomeric signals showed a relative ratio of 1:1 except for the signal at 4.51 ppm. In fact, the peak area for this signal was twice the amount of every other proton anomeric signal, thus indicating the coincidence of H-1 of F with H-1 of G chemical shifts.

By considering all the two-dimensional NMR experiments, the spin systems of all the monosaccharides were identified (Table 2).

**Table 2:** ^1^H and ^13^C NMR assignments of the oligosaccharide OS obtained from acetic acid hydrolysis of the LOS from *P. arcticus* strain 273-4. The spectra were recorded at 310 K at 600 MHz.

Residue	H1 C1	H2 C2	H3 C3	H4 C4	H5 C5	H6 C6	H7 C7	H8 C8	
Lactyl							C1′ ^a^	H2′ ^a^ C2′	H3′ C3′
A α-d-MurNAc^b^	5.36 95.1	3.72 55.0	3.78 78.3	3.66 71.8	3.98 73.5	3.64/3.81 64.5	- 183.2	4.43 79.8	1.40 20.0
B 3,4,6-α-d-Glc*p*	5.16 100.9	3.84 73.6	4.28 78.0	3.98 75.2	4.44 71.4	4.04/4.19 68.8			
C 3-α-l-Rha*p*	5.06 103.5	4.12 68.6	3.86 77.2	3.57 71.7	3.82 70.8	1.30 18.8			
D β-d-Glc*p*	4.95 102.9	3.38 74.8	3.52 77.2	3.43 71.5	3.47 77.1	3.74/3.93 62.2			
E 4-β-d-Glc*p*	4.75 102.2	3.41 74.3	3.67 75.6	3.67 79.7	3.61 76.1	3.82/3.97 61.5			
F 3-β-d-Gal*p*	4.52 104.2	3.66 71.6	3.72 81.6	4.01 69.6	3.75 76.5	3.74/3.92 62.2			
G β-d-Glc *p*	4.51 103.8	3.33 74.4	3.52 77.1	3.41 74.3	3.46 77.3	3.74/3.92 62.2			
H 5-Kdo	n.d.	- 97.8	1.89/2.09 35.5	4.16 67.3	4.11 77.0	3.87 72.8	4.06 70.0	3.78/3.80 64.5	

Additional chemical shifts: ^a^ All lactyl resonances of MurNAc are labelled prime: 1′, carboxylate; 2′, linkage point; 3′, methyl; ^b^ NAc resonances: δ 2.07/23.0 ppm (CH_3_), 175.5 ppm (CO); n.d.: not determined.

**Figure 5:**
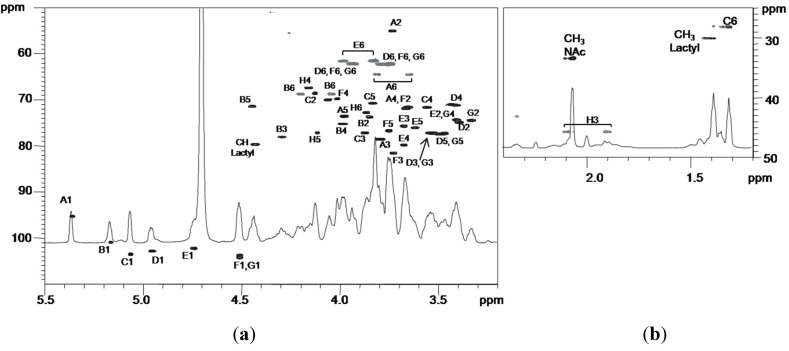
Anomeric/carbinolic (**a**) and aliphatic regions (**b**) of ^1^H-^13^C DEPT-HSQC spectrum of OS core of the LOS from *P. arcticus* strain 273-4*.* The spectrum was recorded in D_2_O at 310 K at 600 MHz.

Residue A with H-1/C-1 signals at δ 5.36/95.1 ppm was identified as a 3-*O*-(1-carboxyethyl) ether of 2-acetamido-2-deoxy glucopyranosyl residue (namely *N*-acetylmuramic acid (NAM)), with an α-anomeric configuration, as suggested by the low ^3^*J*_H-1,H-2_ value (3.1 Hz). Moreover, its H-2 proton at δ 3.72 ppm was correlated, in the DEPT-HSQC experiment (Figure 5), with a C-2 resonance occurring at δ 55.0 ppm, thus indicating a nitrogen-bearing carbon atom. In addition, the HMBC spectrum showed a long range scalar coupling between the signal of H-3 at δ 3.78 ppm with the signal at δ 79.8 ppm, attributed to C-2′ of 1-carboxyethyl substituent. The same experiment also revealed a correlation between the signal at δ 4.43 ppm, attributed to H-2′, with both the signals of C-1′ (δ 183.2 ppm) and C-3′ (δ 20.0 ppm), respectively, of 1-carboxyethyl substituent. Finally, a correlation between H-2 signal at δ 3.72 ppm and the carbonyl signal of NAc group at δ 175.5 ppm was also identified.

The correlations of each H-1 to H-6 with all other protons of residues B, D, E, and G in the TOCSY spectrum provided evidence for the *gluco* configuration of all these ring systems.

**Figure 6:**
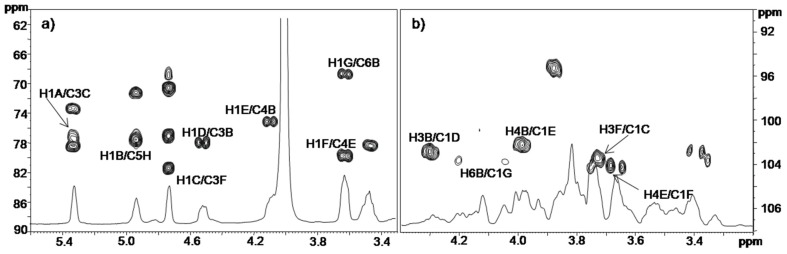
Anomeric (**a**) and carbinolic (**b**) regions of ^1^H-^13^C HMBC spectrum of OS core of the LOS from *P. arcticus* 273-4*.* The spectrum was recorded in D_2_O at 310 K at 600 MHz.

Residue B with H-1/C-1 signals at δ 5.16/100.9 ppm was assigned to a 3,4,6 trisubstituted α-glucose unit on the basis of the small anomeric coupling constant value (^3^*J*_H-1,H-2_ = 3.7 Hz). The downfield shift of C-3, C-4, and C-6 values of this unit at δ 78.0, 75.2, and 68.8 ppm, respectively [27], identified its substitution. This residue was linked to Kdo residue at the *O*-5 position, as shown by the correlation between H-1 B and C-5 of H in the HMBC spectrum (Figure 6, Table 3).

**Table 3:** Correlations for H-1 and C-1 in the two-dimensional ROESY and ^1^H, ^13^C HMBC spectra of the oligosaccharide OS obtained from acetic acid hydrolysis of the LOS from *P. arcticus* strain 273-4. The spectra were recorded at 310 K at 600 MHz.

Anomeric Atom in Sugar Residue (δ)	Correlations to Atom in Sugar Residue (δ)	
	ROESY	HMBC
A H-1 (5.36) C-1 (95.1)	C H-3 (3.86)	C H-3 (3.86)
B H-1 (5.16)	H H-5 (4.11)	H C-5 (77.0)
C H-1 (5.06)	F H-3 (3.72)	F C-3 (81.6)
D H-1 (4.95) C-1 (102.9)	B H-3 (4.28)	B H-3 (4.28)
E H-1 (4.75) C-1 (102.2)	B H-4 (3.98)	B H-4 (3.98)
F H-1 (4.52)	E H-4 (3.67)	E C-4 (79.7)
G H-1 (4.51) C-1 (103.8)	B H-6 (4.04,4.19)	B H-6 (4.04,4.19)

The lack of heptose residue, usually linked in the inner core to the Kdo, has been found so far in the Moraxellaceae [28] and Rhizobiaceae families [29,30]. The only example of a heptose-deficient core region among lipopolysaccharides from psychrophiles was found in *Colwellia psychrerythraea* strain 34H [22].

Residues D and G with H-1/C-1 signals at δ 4.95/102.9 and δ 4.51/103.8 ppm, respectively, were identified as terminal β-glucoses, since none of their carbons were shifted by glycosylation. For both residues the β configuration was inferred by the high ^3^*J*_H-1,H-2_ values (8.1 and 8.0 Hz for D and G, respectively). *Intra*-residue NOE (Nuclear Overhauser Effect) contacts of H-1 with H-3 and H-5 (δ 3.52 and 3.47 ppm, and δ 3.52 and 3.46 ppm, for D and G, respectively) were in agreement with β-anomeric configurations.

A ^3^*J*_H-1,H-2_ coupling constant of 8.0 Hz for residue E indicated a β-configuration, which was also confirmed by *intra*-residue NOEs. The C-4 of residue E was downfield shifted at δ 79.7 ppm with respect to the unsubstituted value [31], thus evidencing that this position was glycosylated. The residue F with H-1/C-1 signals at δ 4.52/104.2 was identified as a *galacto* configured residue since the TOCSY experiment showed correlations only from H-1 to H-4; in particular, it was identified as a β-galactose (^3^*J*_H-1,H-2_ = 8.0 Hz). Moreover, the downfield shift of proton resonance of C-3 at δ 81.6 ppm instead of δ 73.8 ppm of an unsubstituted residue [31] indicated glycosylation at this position.

The residue C with H-1/C-1 signals at δ 5.06/103.5 ppm was recognized as an α-rhamnose residue, since the TOCSY spectrum showed scalar correlations of the ring protons with methyl signal in the up-field region at δ 1.30 ppm. Its α configuration was suggested by the ^3^*J*_H-1,H-2_ value (<3 Hz) and by the value of its C-5 chemical shift [32]. The downfield shift of carbon resonance of C-3 at δ 77.2 ppm with respect to the value of δ 71.0 ppm [31] indicated glycosylation at this position.

Finally, the Kdo (residue H) proton and carbon chemical shifts were identified starting from the diastereotopic protons H-3_ax_ and H-3_eq_ (δ 1.89/2.09 ppm).

The Kdo H-5 proton was identified by vicinal scalar coupling with H-4 in the COSY spectrum. Moreover, the residue resulted to be glycosylated at *O*-5 position, as suggested by the downfield shift of its C-5 carbon signal at δ 77.0 ppm with respect to the value of δ 67.5 ppm for an unsubstituted Kdo [33].

The sequence of the residues was deduced from the HMBC experiment (Figure 6, Table 3) that indicated the following correlations: H-1 of B and C-5 of H, H-3 of B with C-1 of D, H-4 of B with C-1 of E, and both H-6 of B with C-1 of G. In addition, H-1 of rhamnose C displayed a correlation with C-3 of residue F, while C-1 of residue A displayed a correlation with H-3 of C. Finally, H-1 of galactose F displayed a correlation with C-4 of residue E.

*Inter*-residue NOE contacts, obtained from ROESY experiments (Table 3), confirmed this sequence, since dipolar couplings were observed between: H-1 of B and H-5 of H, H-1 of G and both H-6 of B, H-1 of E and H-4 of B, H-1 of F and H-4 of E, H-1 of D and H-3 of B, H-1 of A and H-3 of C, H-1 of C and H-3 of F.

The absolute configuration of residue A is based on NMR considerations. The chemical shift of C-1 at δ 95.1 ppm indicates that the *N*-acetylmuramic acid has the opposite configuration of l-rhamnose, since a value of near 103 ppm would be expected for the same absolute configuration of residue C [34]. As for 1-carboxyethyl substituent, the configuration of (*R*) for C-2′ was deduced by comparing both ^1^H and ^13^C NMR chemical shifts of residue A with those of *N*-acetylisomuramic acid [35,36], characterized by a (*S*) configuration at C-2′.

**Scheme 1:**
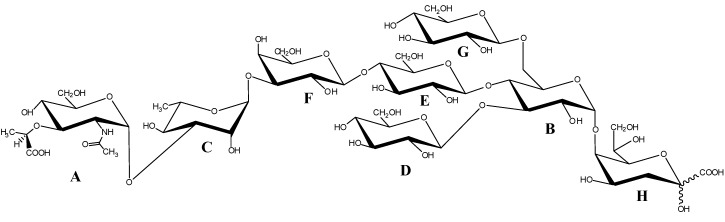
OS core structure of the LOS from *P. arcticus* strain 273-4.

In conclusion, the complete structure of the core oligosaccharide of the LOS from *Psychrobacter arcticus* 273-4 is reported in Scheme 1.

## 3. Experimental Section

### 3.1. Bacteria Growth and LPS Isolation

*P. arcticus* strain 273-4, isolated from permafrost soil located in Siberia. Shake flask cultivation were performed in Luria-Bertani broth [37] at 4 °C in aerobic condition. When the liquid cultures reached late exponential phase (about 90 h, OD _600nm_ 4) cells were collected by centrifugation for 15 min at 7000 rpm at 4 °C.

Dried bacteria cells (3.1 g) were extracted first by PCP method to give very poor yield of LOS, LPS_PCP_ (yield 0.03% *w*/*w* of dried cells) and then by hot phenol/water method [38,39]. A 240 mg amount of water extract was dialyzed (cut-off 3500 Da) and then digested with proteases, DNases, and RNases to remove contaminating proteins and nucleic acids. The sample was dialyzed (cut-off 3500 Da) in order obtaining 96 mg of sample (LPS_W_, yield 3.1% *w*/*w* of dried cells).

### 3.2. Sugar and Fatty Acids Analysis

LOS (1 mg) was treated with HCl/CH_3_OH (1.25 M, 1 mL) and the methanolysis was performed at 80 °C for 16 h. The monosaccharides obtained were acetylated and analyzed as acetylated methyl glycosides by GC-MS. The fatty acids were analyzed as methyl esters [11].

The absolute configuration of the sugars was determinated by gas chromatography of the acetylated (*S*)-2-octyl glycosides [40]*.* All the sample derivatives were analyzed on an Agilent Technologies gas chromatograph 6850A equipped with a mass selective detector 5973N and a Zebron ZB-5 capillary column (Phenomenex, 30 m × 0.25 mm i.d., flow rate 1 mL/min, He as carrier gas). Acetylated methyl glycosides were analyzed using the following temperature program: 140 °C for 3 min, 140 °C → 240 °C at 3 °C/min. Analysis of acetylated octyl glycosides was performed as follows: 150 °C for 5 min, 150 °C → 300 °C at 6 °C/min, 300 °C for 5 min. The temperature program for methyl esters of fatty acids is the following: 140 °C for 3 min, 140 °C → 280 °C at 10 °C/min, 280 °C for 20 min.

### 3.3. Methylation Analysis

The linkage positions of the monosaccharides were determined by GC-MS analysis of the partially methylated alditol acetates (PMAAs).

LOS (1 mg) was methylated with CH_3_I (100 µL) and NaOH powder in DMSO (300 µL) for 20 h [41,42].

To identify the Kdo, the sample was then treated for the reduction of the carboxymethyl groups with sodium boro deuteride NaBD_4_, mildly hydrolyzed (0.1 M trifluoroacetic acid TFA, 100 °C, 30 min) to cleave ketosidic linkages, followed by a reduction (NaBD_4_) of hemiketal group. The product was totally hydrolyzed with 2 M TFA at 120 °C for 2 h, reduced with NaBD_4,_ and acetylated with Ac_2_O and pyridine (50 µL each, 100 °C for 30 min). The mixture was analyzed by GC-MS with the following temperature program: 90 °C for 1 min, 90 °C → 140 °C at 25 °C/min, 140 °C → 200 °C at 5 °C/min, 200 °C → 280 °C at 10 °C/min, at 280 °C for 10 min.

### 3.4. Deacylation of the LOS

The LOS (70 mg) was dried over phosphorus anhydride under vacuum and then incubated with hydrazine (3.5 mL, at 37 °C for 2 h). To precipitate the LOS-OH, cold acetone was added; the pellet was recovered after centrifugation at 4 °C and 7000 rpm for 30 min, washed two times with acetone, and finally suspended in water and lyophilized (55 mg) [43].

### 3.5. Mild Acid Hydrolysis

The LOS (20 mg) was hydrolyzed with 1% aqueous CH_3_COOH (2 mL, 100 °C for 4 h). The resulting suspension was then centrifuged (7500 rpm, 4 °C, 30 min) and the pellet was washed twice with water. The supernatant layers obtained were combined and lyophilized. The mixture of oligosaccharides was then fractionated on a Bio-Gel P-10 column (Biorad, 1.5 × 110 cm, flow rate 15 mL/h, fraction volume 2 mL) and eluted with water buffered with 0.05 M pyridine and 0.05 M AcOH, obtaining the oligosaccharide fraction named OS (6 mg).

### 3.6. Mass Spectrometry Analysis

Electrospray ionization Fourier transform ion cyclotron (ESI FT-ICR) mass spectrometry was performed in negative ion mode using an APEX QE (Bruker Daltonics GmbH, Bremen, Germany) equipped with a 7 Tesla actively shielded magnet. The LOS sample was dissolved at a concentration of ~10 ng/μL, sprayed at a flow rate of 2 μL/min, and analyzed as described previously [44]. Mass spectra obtained were charge-deconvoluted and the mass numbers given refer to the monoisotopic masses of the neutral molecules.

### 3.7. NMR Spectroscopy

^1^H and two-dimensional NMR spectra were performed using a Bruker Avance 600 MHz spectrometer equipped with a cryoprobe (Bruker Italia, Milano, Italy). Two-dimensional homo- and heteronuclear experiments (COSY, TOCSY, ROESY, DEPT-HSQC, and HMBC) were performed using standard pulse sequences available in the Bruker software. ^1^H was measured at 310 K and 318 K while two-dimensional NMR spectra were recorded at 310 K and the mixing time for TOCSY and ROESY experiments was 100 ms. The ^13^C NMR spectrum was recorded in D_2_O at 298 K Bruker Avance 400 MHz spectrometer (data not shown).

## 4. Conclusions

In this paper, the complete structure of the sugar backbone of the LPS from the permafrost isolate *Psychrobacter arcticus* 273-4 is reported.The structure shows a particular inner core region, with a residue of glucose linked to the Kdo in place of a *manno*-heptose. This structural feature has been found only in another psychrophile, namely *Colwellia psychrerythrae* 34H, which showed a mannose residue linked to the Kdo.

Generally, the oligo- and polysaccharides produced by marine bacteria are distinguished by the acidic character [45] and by the occurrence of unusual sugars [46], non-sugar substituents [22,47,48,49] or structures that are highly phosphorylated [10]. Although *P. arcticus* 273-4 was isolated from Arctic permafrost, it displays similar characteristics of cold-adapted marine isolates, due to the presence of the unusual residue of NAM. *N*-acetylmuramic acid, commonly encountered as a component of bacterial cell-wall peptidoglycan, has been already found in the *O*-specific polysaccharide of *Yersinia ruckerii* [50] and *Proteus penneri* [51], but to the best of our knowledge, this is the first time that it has been found in a core oligosaccharide.

It is well known that cold-adapted microorganisms are able to modify the fluidity of the cellular membrane in response to a lowering of temperature by producing a higher content of unsaturated, polyunsaturated, and methyl-branched fatty acids [52,53]. Instead, how bacteria modify the LPS structures in response to the cold stress is still poorly understood.

Even though only few LPS structures from cold-adapted bacteria have been characterized [11,12,22,23], their attractive feature is the production of rough lipopolysaccharides. Moreover, it is worth noting that *Psychrobacter arcticus* 273-4, a permafrost isolate, shares this feature with marine isolates. To the best of our knowledge, only two examples of smooth lipopolysaccharides isolated from psychrophiles have been reported so far [54,55], even if the isolates were grown at 24 °C.

By increasing the number of characterized LPS structures from psychrophiles, it will be conceivable in the future to find a connection between the lack of the polysaccharidic portion and the Gram-negative membrane cold adaptation.
